# Periodontal Pathogens Inhabit Root Caries Lesions Extending beyond the Gingival Margin: A Next-Generation Sequencing Analysis

**DOI:** 10.3390/microorganisms9112349

**Published:** 2021-11-13

**Authors:** Shoji Takenaka, Naoki Edanami, Yasutaka Komatsu, Ryoko Nagata, Traithawit Naksagoon, Maki Sotozono, Takako Ida, Yuichiro Noiri

**Affiliations:** 1 Division of Cariology, Operative Dentistry and Endodontics, Faculty of Dentistry & Graduate School of Medical and Dental Sciences, Niigata University, Niigata 951-8514, Japan; edanami@dent.niigata-u.ac.jp (N.E.); lemmings@dent.niigata-u.ac.jp (R.N.); naksagoon-ttw@dent.niigata-u.ac.jp (T.N.); sotozono@dent.niigata-u.ac.jp (M.S.); tida@dent.niigata-u.ac.jp (T.I.); noiri@dent.niigata-u.ac.jp (Y.N.); 2 Division of Periodontology, Faculty of Dentistry & Graduate School of Medical and Dental Sciences, Niigata University, Niigata 951-8514, Japan; komatsu@dent.niigata-u.ac.jp

**Keywords:** root caries, oral microbiota, active lesion, MiSeq amplicon sequencing

## Abstract

We performed a comprehensive microbiome analysis of root caries lesions using 22 teeth extracted from patients with severe periodontitis. The carious lesions were mechanically collected and cryo-pulverized following tooth extraction. Differences in the microbiome were compared between independent lesions at the supragingival site (SG) and lesions extending beyond the gingival margin (GCB). DNA was extracted and the microbiome was characterized on the basis of the V3-V4 hypervariable region of the 16S rRNA gene using paired-end sequencing on an Illumina MiSeq device. The microbiota in root caries lesions showed compositionally distinct microbiota depending on the location. The most abundant OTUs in the SG group were *Streptococcus* (26.0%), *Actinomyces* (10.6%), and *Prevotella* (7.6%). GCB presented *Prevotella* (11.1%) as the most abundant genus, followed by *Fusobacterium* (9.6%) and *Actinomyces* (8.7%). The SG group showed a lack of uniformity in microbiota compared with the GCB group. The bacterial profiles of GCB varied considerably among patients, including periodontal pathogens such as *Porphyromonas*, *Selenomonas*, *Filifactor*, *Peptococcus*, and *Tannerella*. Periodontal pathogens inhabit root caries lesions that extend beyond the gingival margin. This study provides a new perspective for elucidating the microbial etiology of root caries.

## 1. Introduction

Dental caries has been increasing in geriatric populations because of the higher retention rate of natural teeth. Root caries, defined as a progressive lesion found on the tooth root, has become a major concern [1]. The prevalence of root caries increases with age [2], and it occurs even among healthy older adults [3]. A recent systematic review showed that individuals who are older, have a lower socio-economic status or use tobacco, as well as those with more root caries experience, gingival recession, and poor oral hygiene, have a higher risk of developing new root caries [4].

Root caries initiates on exposed root surfaces due to gingival recession. Since gingival recession can occur as a result of periodontitis and periodontal therapy, systemic and local interventions are needed to prevent gingival recession and root caries in periodontal patients [5,6]. Examples of interventions include dietary control, oral hygiene instruction, the use of a fluoride dentifrice, stimulation of salivary flow, oral biofilm control, and local application of a concentrated fluoride varnish [5]. The progression of root caries is accompanied by acid decalcification of the inorganic component (hydroxyapatite) and protein decomposition of the organic component (collagen) of tooth structure [7].

Conventional culture-based studies have frequently detected streptococci, lactobacilli, and the genus *Actinomyces* in root caries, especially in active lesions; therefore, these bacteria have been implicated in its initiation and progression [8,9,10,11,12]. These acidogenic and aciduric organisms can synergize to enhance the production of extracellular polysaccharides and promote further acidification [13]. However, molecular-based studies have demonstrated that these strains may play a limited role as pathogens of root caries, the microbiota of which are dominated by various genera, including *Pseudoramibacter, Veillonella*, *Enterococcus*, *Selenomonas*, *Propionibacterium, Atopobium*, and *Corynebacterium* [14,15]. Thus, the microbial etiology of root caries may be more complicated than previously reported.

Recently, next-generation DNA sequencing (NGS) methods have been used to comprehensively analyze the microbiome of dental caries. Although biofilm samples are often taken from root caries lesions [16,17,18], studies on the microbiome of root caries are limited [19]. Since the bacterial profiles of carious root dentin differed in diversity and bacterial dominance from biofilm on the lesions [14], it is necessary to distinguish the microbiota of root caries from that of dentin caries to elucidate the microbial etiology.

The purpose of this study was to perform a comprehensive analysis of the microbiome in entire root caries lesions using teeth extracted from patients with severe periodontitis. In this study, we compared the differences in the microbiome relative to the location of root caries. The lesions on the root surface remained localized or extended beyond the gingival margin. The statistical null hypothesis was that the microbiome in the root caries lesion expanding across the gingival margin would not show an increase bacterial diversity.

## 2. Materials and Methods

### 2.1. Study Population

A total of 23 participants (16 women, 7 men; mean age, 67 years; range, 59–79 years) with severe periodontitis (Stage III, Grade B) [20] who had root caries and needed tooth extraction were selected from patients attending the dental clinic at Niigata University Medical and Dental Hospital in Japan. The teeth included in this study were sufficiently mobile to allow forceps extraction without the use of dental elevators. All samples contained an active lesion, which felt soft or leathery on probing with the CPI probe [21,22]. The inclusion criteria were as follows: (a) presence of one site with ≥6 mm probing pocket depth or presence of clinical attachment loss more than 5 mm; (b) presence of one site with radiographic bone loss exceeding 1/3 of the root length; (c) presence of bleeding on probing. The main exclusion criteria included: (a) prior periodontal surgery; (b) patients who had taken local and/or systemic antibiotics within the previous four weeks [23]; (c) patients who had used antiseptic mouthwash in the last four weeks; (d) smoker; (e) pregnant or lactating women. All subjects were asked to avoid eating or drinking 2 h before sampling [23]. Analgesia was induced by local injection of lidocaine (29 mg/mL) and adrenaline (0.0125 mg/mL) (Xylocaine Cartridge for Dental Use, Dentsply Sirona, Tokyo, Japan). The study protocol was approved by the Niigata University Ethics Committee (approval number 2016-0022), and the methods were carried out in accordance with the approved guidelines. All participants signed an informed consent form before participating in the study.

The tooth was extracted while avoiding the root caries. The tooth was then gently rinsed with saline to remove any excess blood or loose debris. Excess soft tissue was carefully removed with a scalpel, and the superficial dental biofilm on the carious lesion was thoroughly removed using sterile cotton balls dampened with sterile distilled water. The dentin caries lesions were collected using a sterile spoon excavator or a round bur at a slow speed [14]. All the samples were obtained by one of the authors, placed in phosphate-buffered saline (PBS), and stored at −20 °C.

### 2.2. Histological Observation

To examine the histopathology of root caries, a non-decalcified section of root caries was prepared as described previously, with modifications [24]. Briefly, the teeth were fixed in 10% buffered formalin. Following dehydration in a graded series of ethanol (70–100%) and infiltration with isopropanol and epoxy resin, the samples were embedded in epoxy resin and polymerized. The polymerized blocks were cut in a mesio-distal direction using a hard tissue cutting machine (BS-300CL, EXAKT, Norderstedt, Germany). The undecalcified sections were ground to a thickness of approximately 30 µm using a grinding machine (MG-400CS, EXAKT). The sections were stained with Hematoxylin and eosin (H&E).

### 2.3. Sample Collection and DNA Extraction

The collected dentin shavings were freeze-dried (VD-250R Freeze Dryer, TAITEC, Saitama, Japan) and pulverized at 1500 rpm for 2 min (Shake Master Neo, Bio Medical Science, Tokyo, Japan). The powdered root caries samples were added to a lysis solution (Lysis Solution F, Nippon Gene, Tokyo, Japan) and left to stand for 10 min at 65 °C. The samples were centrifuged at 12,000× *g* for 1 min, and genomic DNA was extracted using an MPure Bacterial DNA Extraction Kit (MP Biomedical, Santa Ana, CA, USA).

### 2.4. Microbiome Analysis

The samples were divided into two experimental groups: root caries that existed independently above the gingival margin (SG), and root caries that progressed below the gingival margin (GCB). The bacterial microbiota was investigated by targeted 16S rRNA gene (V3-V4 region) sequencing using the Illumina MiSeq system (2 × 300 bp paired-end reads) (Illumina, San Diego, CA, USA) as described previously with some modifications [25,26] (Bioengineering Lab. Co. Ltd., Kanagawa, Japan). A two-step polymerase chain reaction (PCR) was performed to generate amplicon libraries. Ambiguous bases, low-quality reads, and sequences with read lengths below 200 bp were discarded. The remaining sequences were clustered into phylotypes using QIIME2, with a minimum coverage of 99% and a minimum identity of 97%. A representative sequence for each operational taxonomic unit (OTU) was selected for taxonomic assignment with reference to the EzBioCloud 16S database. Protocols for amplicon libraries have been described previously [25,26].

### 2.5. Statistical Analysis

Demographic characteristics were compared using the paired *t*-test, Wilcoxon rank-sum test, or Pearson’s chi-squared test. The analysis was based on the relative abundance of each taxonomic group, and the results were reconstructed for this study. The Chao 1 index and Shannon index were compared using the Wilcoxon rank-sum test. Analyses were performed using SPSS version 28 (IBM Corp., Armonk, NY, USA). The bacterial characteristics at the phylum and genus levels were compared with Lefse analysis. PERMANOVA was performed for beta diversity. Statistical significance was set at *p* < 0.05.

## 3. Results

### 3.1. Demographic and Clinical Characteristics

Demographic and clinical characteristics of the participants are shown in Table 1. No significant differences were observed between SG and GCB.

### 3.2. Histopathology of Root Caries

**Figure 1:**
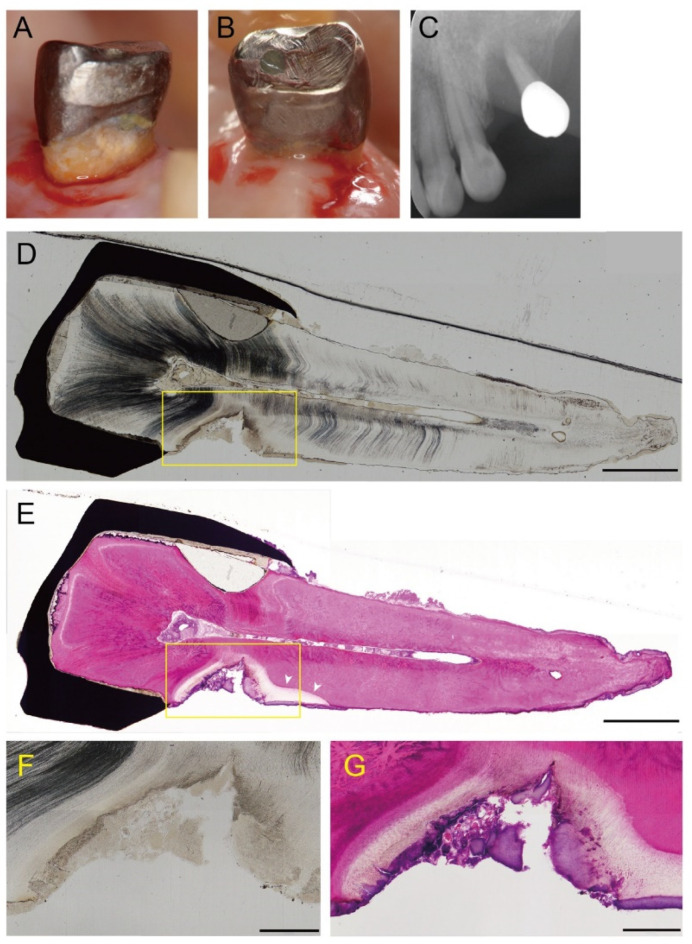
A representative case with active supragingival root caries. Tooth 24 in a 79-year-old woman had a root caries with severe periodontitis. The mesial (**A**) and a distal aspect (**B**) before tooth extraction. An active lesion can be observed at the mesial root surface. (**C**) X-ray photograph. The tooth shows advanced bone resorption due to severe periodontitis. (**D**) A longitudinal section through a cavitated lesion of an active root caries (original magnification ×4). The lesion progresses similar a notch and extends to 2/3 of the dentin towards the pulp. Scale bar = 2 mm. (**E**) H&E staining of a longitudinal section (original magnification ×4). The dentin shows extensive demineralization close to the dental pulp. Demineralization has progressed to just below the cementum on the apical side (arrowheads). Scale bar = 2 mm. (**F**,**G**) Higher magnification of the area is indicated by the squares in D and E, respectively (original magnification ×10). Scale bar = 500 µm. (**F**) The dentine structure is irregular, partially destroyed, and exhibits a non-tubular morphology. (**G**) The dentin underlying the biofilm is extensively demineralized.

A representative longitudinal section of a cavitated lesion is shown in Figure 1. Tooth 24 in a 79-year-old woman contained root caries and presented severe periodontitis at the mesial surface, with advanced bone resorption (Figure 1A,C). The lesion progressed similar to a notch and extended to the pulp side through 2/3 of the dentin (Figure 1D). H&E staining showed that the dentin was extensively demineralized close to the dental pulp (Figure 1E). Demineralization also progressed to just below the cementum on the apical side (arrowheads), although the structure of the cementum remained intact. A higher magnification view of the lesion showed that the dentin structure was irregular, partially destroyed, and exhibited a non-tubular morphology (Figure 1F). The dentin underlying the biofilm was extensively demineralized (Figure 1G).

### 3.3. Alpha Diversity Analyses of the Bacterial Community

**Figure 2:**
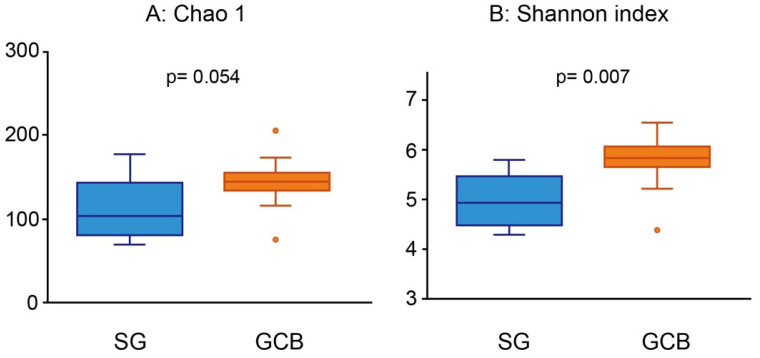
Alpha diversity analysis using Chao 1 (**A**) and Shannon index (**B**). The root caries lesions collected from 22 participants are divided into two groups according to the location of the lesion. Each value is presented as a box plot. The top, middle, and bottom lines of the boxes represent the 25th, 50th (median), and 75th percentiles, respectively. The significance of differences between the two groups is evaluated. *p* values < 0.05 are considered to indicate statistical significance. SG: root caries that existed independently above the gingival margin, GCB: root caries that progressed below the gingival margin.

The average number of OTUs was 112.9 ± 38.2 for SG, and 142.36 ± 30.2 for GCB. A bacterial diversity analysis was performed to characterize the microbes in the root caries lesion. GCB showed relatively higher richness than SG, whereas there was no significant difference in the Chao 1 index (*p* = 0.054, Figure 2A). The Shannon diversity index of SG (5.0 ± 0.5) was significantly lower than that of GCB (5.8 ± 0.6) (*p* = 0.007, Figure 2B), indicating the lack of uniformity of microbiota in the SG group.

### 3.4. Differentiating Composition of SG and GCB

**Figure 3:**
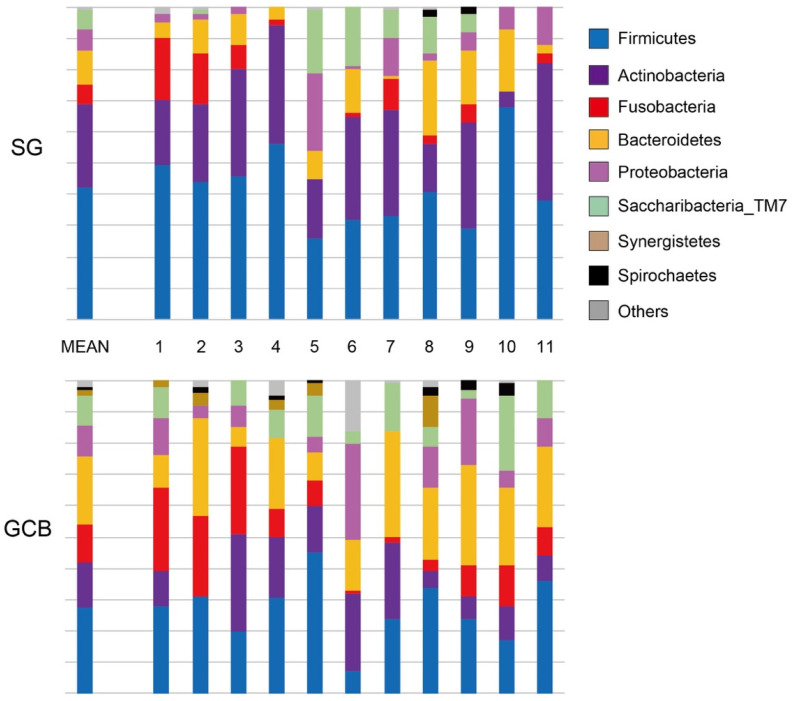
Distribution pattern of 8 phyla in root caries lesions. SG: root caries that existed independently above the gingival margin, GCB: root caries that progressed below the gingival margin.

The microbial composition of the root caries lesions was analyzed by 16S rRNA sequencing. The distribution pattern of the eight phyla, which accounted for more than 1% of the total, is shown in Figure 3. Firmicutes was the most prevalent phylum in both groups; however, the abundance of SG was at least 4.5 times higher than that of GCB (*p* < 0.05, linear discriminant analysis effect size (LEfSe)). Similarly, Actinobacteria was 4.5 times higher in the SG group than in the GCB group (*p* < 0.05). Conversely, Bacteroides in the GCB group was 4.2 times higher than that in the SG group (*p* < 0.05).

**Figure 4:**
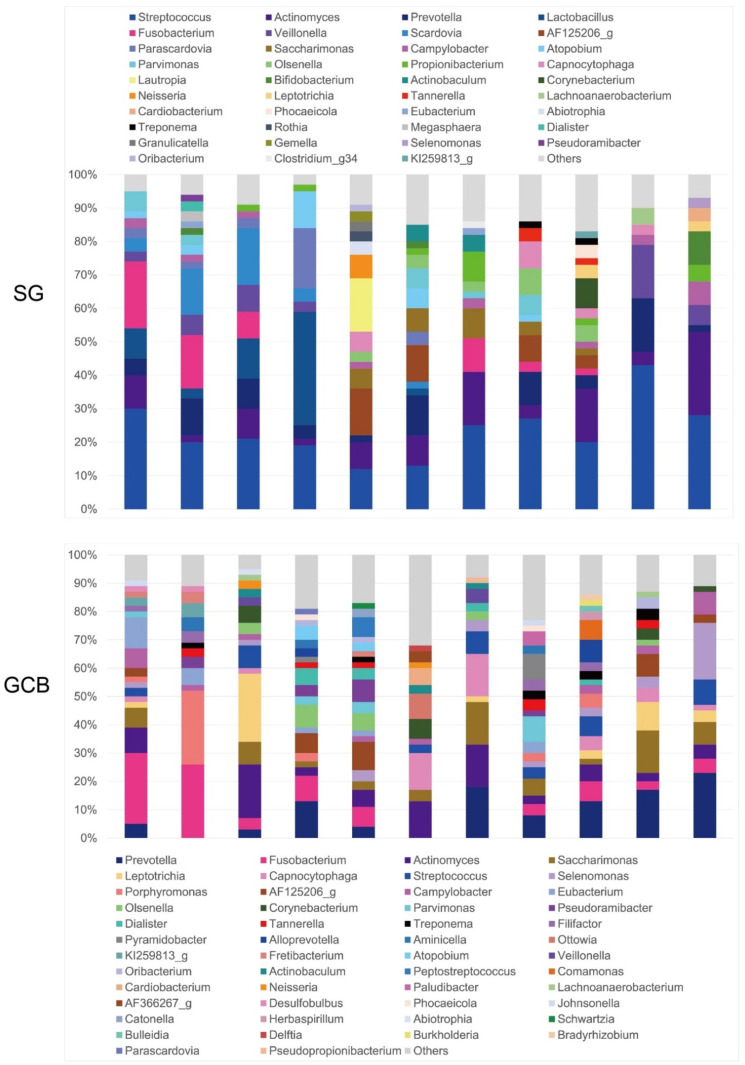
Relative abundance of major bacterial genera. Sequence data for determining the genera in the SG and GCB are obtained from 11 individual root caries lesions. SG: root caries that existed independently above the gingival margin, GCB: root caries that progressed below the gingival margin.

At the genus level, 19 genera in SG and 24 genera in GCB were present at a rate of >1% (Figure 4). The most abundant OTUs in the SG group were *Streptococcus* (26.0%), followed by *Actinomyces* (10.6%), *Prevotella* (7.6%), and *Lactobacillus* (6.1%). GCB presented *Prevotella* (11.1%) as the most abundant genus, followed by *Fusobacterium* (9.6%) and *Actinomyces* (8.7%). The bacterial profiles of the GCB varied according to the subjects and were more complex than those of the SG with inconsistent features. On average, the genus *Streptococcus* comprised only 4.5% of the genera in the GCB group. Although the genus *Lactobacillus* was detected in the SG group at an average rate of 6%, it was not detected in the GCB group.

**Figure 5:**
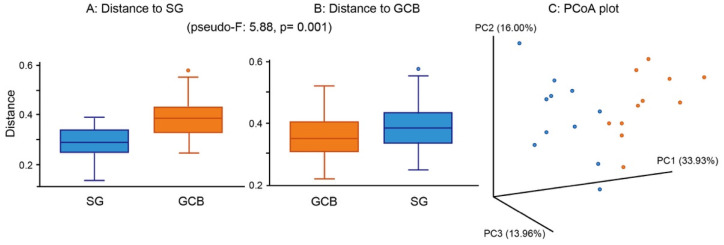
β-diversity of root caries lesions. Distance boxplot (**A**,**B**) and PCoA plot-weighted unifrac (**C**). The analysis shows the significantly distinguished clustering between SG and GCB. Statistical result appears in the figure. SG: root caries that existed independently above the gingival margin, GCB: root caries that progressed below the gingival margin.

When comparing the groups in terms of β-diversity, considering the distance and dissimilarity among samples, the results showed a clearly distinguished clustering of microbial communities according to the location (Figure 5A,B). There was a significant difference in the β-diversity between the groups, suggesting that the bacterial composition was altered by the location of the root caries lesion. A principal component analysis showed that individual SGs were clearly separated from individual GCBs (Figure 5C), indicating that the bacterial compositions of the two groups were dissimilar.

**Figure 6:**
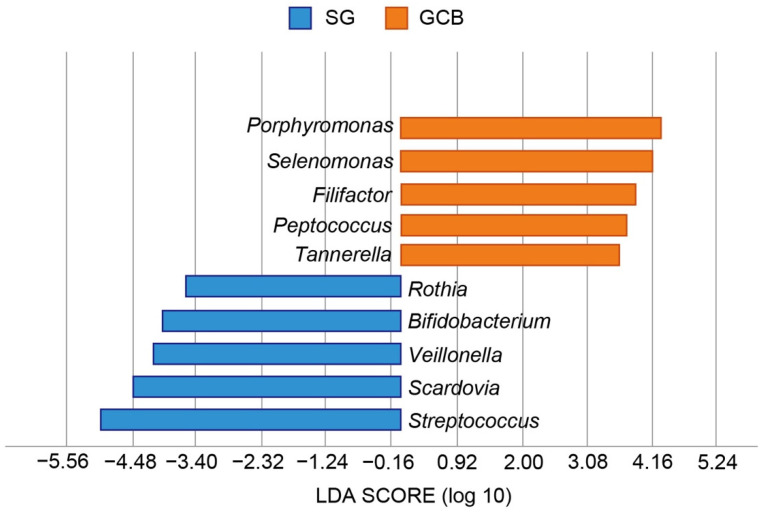
Graphics of Linear discriminant analysis effect size (LEfSe) for SG and GCB. Horizontal bars represent the effect size for each taxon. The length of the bar represents the log 10 transformed LDA score, indicated by vertical dotted lines. The threshold on the logarithmic LDA score for discriminative features is set to 3.0. The taxon of bacteria with statistically significant change (*p* < 0.05) in the relative abundance is expressed alongside the horizontal lines. SG: root caries that existed independently above the gingival margin, GCB: root caries that progressed below the gingival margin.

Lefse revealed the bacterial characteristics of each group at the genus level (Figure 6). In the SG group, the genera *Streptococcus*, *Scardovia*, *Veillonella*, *Bifidobacterium*, and *Rothia* were at least three-fold higher than those in the GCB group (*p* < 0.05). Conversely, in the GCB group, the genera *Porphyromonas*, *Selenomonas*, *Filifactor*, *Peptococcus*, and *Tannerella* were at least three-fold higher than those in the SG group (*p* < 0.05).

## 4. Discussion

Root caries lesions are most commonly located on the exposed root surface, as well as the gingival margin, the margin of previous restoration, and the cementoenamel junction [6]. When the root caries lesion is close to the gingival margin or extends into the subgingival site, gingival crevicular fluid (GCF) can modulate the microbial composition because of its neutral to weakly alkaline pH properties.

In this study, we investigated whether the location of a root caries lesion influenced the composition of the microbiota using NGS. The findings rejected the null hypothesis that the microbiome in root caries lesions expanding across the gingival margin would not show an increase bacterial diversity. Sequencing analysis of the 16S rRNA gene revealed that SG and GCB had compositionally distinct microbiota, specifically that GCB had more diverse microbiota than SB (Figure 2). The SB group showed a low-homogeneous flora in which certain bacteria predominated. The largest difference in characteristic features was the ratio of the genus *Streptococcus*, in which the SG group was 4.5 times higher than the GCB group (Figure 6). Conversely, many periodontal pathogens, such as the genera *Porphyromonas* and *Tannerella*, were detected in the GCB group (Figure 6). Potential pathogens involved in the onset and progression of periodontal disease, such as *Selenomonas* and *Filifactor*, were also detected [27]. GCF may play a modulatory role in the acidity of the biofilm [7], creating a favorable environment for periodontal pathogens.

This phenomenon can be explained by the buffering capacity of the GCF. Acidogenic and aciduric bacteria, such as *Lactobacillus* and *Bifidobacterium* species, have been frequently detected in root caries lesions [12,20,28] and dentin caries [29]. The prevalence of *Lactobacillus* spp. in carious lesions increases as pH decreases [30]. Kianoush et al. reported the effect of pH on the dentin microbiome [23]. Increased *Lactobacillus* levels were associated with a lower pH, specifically pH 4.5–5.0 (*p* = 0.0003). In this study, *Lactobacillus* spp. were present only in the SB group, but were not detected in the GCB group. GCF might neutralize acidic conditions in the biofilm and alter the environment in the root caries lesion. *Prevotella* spp. were the most abundant in the GCB, accounting for 11.1% on average. This species has been detected at higher pH levels, specifically pH 5.5–6 [31]. Taken together, when the lesion extends into the subgingival margin, the biofilm underlying the root caries will be affected by the subgingival environment, resulting in a modulation of the microbiota.

In the present study, *Scardovia* spp. were significantly more abundant in the SG group (Figure 6). *S. wiggsiae* has been detected in caries in children and adolescents and has thus been considered a caries-associated microorganism [32]. In a study by Damé-Teixeira et al., *S. wiggsiae* and *S. inopinata* were detected in root caries [33], and these strains exhibited high acid production and tolerance to lactic acid by a unique metabolic pathway known as “Bifid shunt” [32]. A positive correlation was reported between the metabolic abundance of *Lactobacillus* spp., *Bifidobacteriaceae* members, and *Scardovia* in root caries [33]. These findings suggest that the root lesion in the SG group is located in an acidic environment, where acidogenic and aciduric bacteria predominate.

In culture-based studies, *Actinomyces* species remained dominant in active root caries [10,11,28]. These species may play an important role in controlling mineral loss through pH-modulating mechanisms [34]. *Actinomyces* species were also predominant in this study; however, the average abundance was only 10.6% in the SB group and 8.7% in the GCB group. Dame-Teixeira et al. performed a gene expression analysis of *Actinomyces* species in the microbiota of root surfaces with and without root caries using RNA-seq [35]. The results showed that *Actinomyces* species did not enhance gene expression in carious root biofilms. These species may exist as commensal members in root surface sites, metabolizing sugars and saving energy, rather than promoting root caries. In fact, a molecular biology-based study detected a wide variety of bacterial species, including *Streptococcus mutans*, *Actinomyces*, and *Lactobacillus* species, in root caries [14].

This is the first pilot report to analyze the microbiota in entire root caries lesions, by location, using MiSeq amplicon sequencing. Most studies targeted the biofilm overlying the lesion or dentin sample, collected by a sterile spoon excavator or a round bur in clinical practice. However, the detection and diagnosis of root caries lesions are complex, due to variations in color and texture [6]. In fact, demineralization progressed just below the cementum on the apical side, although the structure of the cementum remained intact (Figure 1E). Thus, tooth extraction is the best way to comprehensively analyze the microbiome in root caries lesions.

However, this study had some limitations, including a small sample size. Only 11 participants per study group were included in this study. The bacterial profiles of GCB showed considerable variation among subjects, revealing inconsistent features (Figure 4). The microbiota of GCB is affected by the periodontal environment just below root caries. In addition, the subgingival microflora changes following periodontal interventions such as scaling and root planning (SRP) and chemotherapy [36]. SRP is effective in decreasing the relative abundance of periodontitis-associated genera in the subgingival plaque, such as *Porphyromonas*, *Treponema*, *Tannerella*, and *Prevotella* [36], altering the root surface morphology [37]. Simultaneously, *Streptococcus* and *Actinomyces* increase and become the dominant genera after intervention.

Probiotics, a type of bacteriotherapy, may support SRP by providing a general improvement in clinical indexes and reduction in periodontopathogens [38,39]. Since these adjunctive agents may also affect the microbiome in root caries, these factors including periodontal status and interventions should be taken into consideration in future studies.

Another limitation is that this study targeted only active root lesions. There seems to be variation in the microbiota between active and non-active lesions, although differences between active and arrested cavities have not yet been elucidated [7]. Beighton et al. investigated the numbers of *Streptococcus mutans*, lactobacilli, yeasts, and Gram-positive pleomorphic rods according to lesion status using a microbiological culture method [21]. The results showed that lactobacilli and *S. mutans* predominated in soft and leathery lesions. In addition, Nicolae et al. reported a causal relationship between salivary parameters and caries activity [40]. The mean values of calcium phosphates and pH were statistically higher in caries-resistant subjects when compared with caries-active subjects. Taken together, further microbiome studies according to the lesion status, location, and periodontal status are expected.

Although the GCB includes some periodontal pathogens, it is unclear how these bacteria contribute to collagen matrix degradation during caries progression. Matrix metalloproteinases (MMPs), which are directly involved in dentin matrix degradation during caries formation, show activity under neutral conditions [7]. The pH modulation by gingival crevicular fluid may accelerate protein degradation. Further investigations with careful consideration in experimental design are needed to elucidate the microbial etiology of root caries. Detailed studies, such as transcriptome, proteomic, and functional analyses, are required.

## 5. Conclusions

Within the limitations of this study, the microbiota in root caries lesions showed compositionally distinct microbiota depending on the location. The microbial profile in the carious lesion extending beyond the gingival margin was more diverse and complex than that in the supragingival site. Periodontal pathogens inhabit root caries lesions that extend into the gingival margin. This study provides a new perspective for elucidating the microbial etiology of root caries.

## Figures and Tables

**Table 1:** Demographic and clinical characteristics of study subjects.

	SG (n = 11)	GCB (n = 11)	*p* Value
Age (mean ± SD)	66.7 ± 5.2	69.0 ± 5.5	0.474
Gender female (%)	72.7	63.6	0.748
Number of remaining teeth (mean ± SD)	19.5 ± 5.7	18.9 ± 5.6	0.847
Root caries teeth (mean ± SD)	2.5 ± 2.2	2.7 ± 1.7	0.748
O’ Leary’s plaque control record (%)	27.43 ± 8.05	29.82 ± 8.21	0.401

SG: root caries that existed independently above the gingival margin, GCB: root caries that progressed below the gingival margin.

## Data Availability

The data sets used during the study are available from the corresponding author upon reasonable request.
